# Distinguishing intrinsic photon correlations from external noise with frequency-resolved homodyne detection

**DOI:** 10.1038/s41598-020-79686-0

**Published:** 2020-12-29

**Authors:** Carolin Lüders, Marc Aßmann

**Affiliations:** Experimentelle Physik 2, Technische Universität Dortmund, 44221 Dortmund, Germany

**Keywords:** Polaritons, Quantum optics, Ultrafast photonics

## Abstract

In this work, we apply homodyne detection to investigate the frequency-resolved photon statistics of a cw light field emitted by a driven-dissipative semiconductor system in real time. We demonstrate that studying the frequency dependence of the photon number noise allows us to distinguish intrinsic noise properties of the emitter from external noise sources such as mechanical noise while maintaining a sub-picosecond temporal resolution. We further show that performing postselection on the recorded data opens up the possibility to study rare events in the dynamics of the emitter. By doing so, we demonstrate that in rare instances, additional external noise may actually result in reduced photon number noise in the emission.

## Introduction

Photon correlation measurements are a central backbone of many applications relying on quantum technologies. They are employed frequently both as a characterization tool and as a part of communications protocols. For purposes of characterization, they are fundamental for tasks as different as determining the single photon purity of single photon sources^1^, monitoring the stable operation of lasers^2^ and investigating diffusion via fluorescence correlation spectroscopy^3^. For communications protocols, they play a prominent role in monitoring quantum light sources to enable secret key distillation^4^ as well as in advanced protocols that merge chaos communication and ghost imaging^5^. Especially the latter applications require a constant real time monitoring of photon correlations. In most cases, the equal time correlation function $g^{(2)}(0)$ is the central quantity of interest. It is given by

1 $$\begin{aligned} g^{(2)}(0)=\frac{\langle {\hat{a}}^\dagger {\hat{a}}^\dagger {\hat{a}} {\hat{a}} \rangle }{\langle {\hat{a}}^\dagger {\hat{a}} \rangle ^2}, \end{aligned}$$

where ${\hat{a}}^\dagger$ and ${\hat{a}}$ denote the photon creation and annihilation of the light field mode of interest, respectively. It corresponds to the relative probability to detect photon pairs from the light source, normalized to a light source of the same intensity that emits statistically completely independent photons. It is thus a measure of the relative variance of the photon number distribution and therefore of photon number noise.

There are different ways to realize photon correlation experiments. For experiments that focus on single photons, many of them operate in the regime of discrete variables, so they rely on photon counting. The most commonly realized experiment in this respect is the Hanbury Brown–Twiss setup^6^, which utilizes two detectors and correlates their output. This experiment typically employs photo diodes^7^, which are usually limited to a temporal resolution of hundreds of picoseconds if good detector quantum efficiency is required. In order to achieve better temporal resolution, superconducting nanowire detectors may be able to enhance the temporal resolution to few picoseconds^8^ or one may instead utilize streak cameras^9–11^, two-photon absorption^12^, up-conversion^13^ or transition edge sensors^14^.

However, photon-counting experiments are not only sensitive to the intrinsic photon number noise of the light field, but may also detect other noise sources, e.g. mechanical vibrations or thermal effects that modify the efficiency of coupling the light field to the detector, stray light entering the detector or air turbulence acting on free-space light beams. All of these external influences may make the light field look noisier than it actually is and will in most cases increase the value of $g^{(2)}(0)$ found in measurements from the intrinsic value of $g^{(2)}(0)$ given by the photon statistics of the light field. The correlation measurements outlined above usually require quite long integration times to produce a single value of $g^{(2))}(0)$, which results e.g. in integration times of minutes for state of the art real-time monitoring of quantum light sources in quantum key distribution^4^. Accordingly, it is very difficult to distinguish between intrinsic and external noise contributions to $g^{(2)}(0)$ in such experiments.

Photon counting is not the only way to measure $g^{(2)}(0)$. Continuous variable approaches, e.g. based on phase-averaged sampling of the light field quadratures using homodyne detection require a local oscillator, but are known to be reliable and efficient, especially for light fields that are not Fock states^15–18^. In contrast to photon counting approaches, it is already possible to perform measurements of $g^{(2)}(0)$ in real time using homodyne detection. Measurement rates of 100 kHz have already been reported^20^.

Here, we demonstrate that such fast measurements of $g^{(2)}(0)$ open up the possibility to unambiguously distinguish between intrinsic and external photon number noise as long as the typical timescales on which those fluctuations occur differ significantly. As the intrinsic time scale of photon number fluctuations is given by the coherence time of the light field, which typically ranges between picoseconds and nanoseconds, while mechanical vibrations take place rather on the scale of milliseconds to seconds, this condition is usually well fulfilled. We further show that such fast measurements may open up the possibility to identify rare events and demonstrate this capability by investigating different kinds of fluctuations in a steady state polariton condensate.

## Real-time homodyne detection

**Figure 1:**
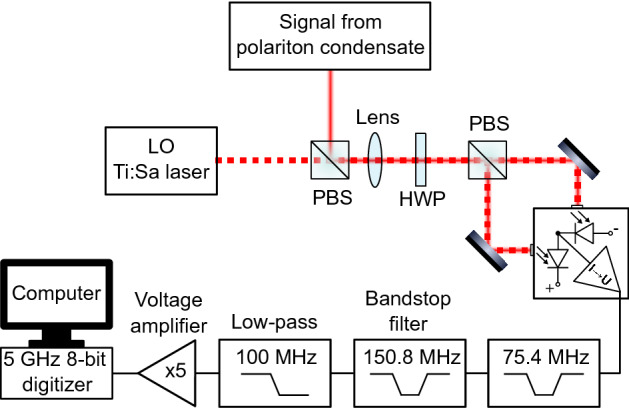
Schematic of the homodyne detection setup. LO: local oscillator; PBS: polarizing beam splitter; HWP: half-wave plate.

In a typical homodyne experiment, the signal of interest is superposed with a strong light field, the local oscillator, on a beam splitter, as depicted in Fig. 1.

These light fields are represented by operators ${\hat{a}}_{LO}$ and ${\hat{a}}_S$, respectively. The light fields at the two output ports of the beam splitter then correspond to the sum (+) and the difference (−) of the local oscillator and the signal field:

2 $$\begin{aligned} {\hat{a}}_{\pm }=\frac{1}{\sqrt{2}}\left( e^{i \phi } {\hat{a}}_{LO} \pm {\hat{a}}_S \right) , \end{aligned}$$

where $\phi$ corresponds to the relative phase between the signal and the local oscillator. The two fields are then detected on two separate photodiodes and the difference signal of the two detector outputs is recorded, which corresponds to

3 $$\begin{aligned} {\hat{Q}}={\hat{a}}_+^\dagger {\hat{a}}_+ - {\hat{a}}_-^\dagger {\hat{a}}_- =\langle {\hat{a}}_{LO}\rangle \left( e^{i\phi } {\hat{a}}^\dagger _S +e^{-i\phi } {\hat{a}}_S \right) . \end{aligned}$$

Here $\langle {\hat{a}}_{LO}\rangle$ approximately becomes $\sqrt{n}_{LO}$ if the local oscillator is an intense and coherent beam with a known mean photon number of $n_{LO}$. This term is directly proportional to the phase-sensitive field quadrature ${\hat{q}}_\phi$ of the signal light field. It is now possible to calculate $g^{(2)}(0)$ from the moments of the quadrature distribution:

4 $$\begin{aligned} \langle Q^2 \rangle&= n_{LO} \left( 2\langle {\hat{a}}^\dagger _S {\hat{a}}_S\rangle +1 \right) \end{aligned}$$

5 $$\begin{aligned} \langle Q^4 \rangle&= n_{LO}^2 \left( 6\langle {\hat{a}}^\dagger _S {\hat{a}}^\dagger _S {\hat{a}}_S {\hat{a}}_S \rangle +12\langle {\hat{a}}^\dagger _S {\hat{a}}_S\rangle +3\right) \end{aligned}$$

However, it should be noted that these averages only hold for the case of phase-averaged measurements.

Let us now discuss the different timescales present in homodyne experiments and how they open up the possibility to distinguish between intrinsic and external photon number noise. First, the signal light field is not static, but its phase or amplitude may change randomly on a timescale given by its coherence time. Accordingly, it is an important prerequisite for an adequate quadrature measurement that the duration of the local oscillator does not exceed the coherence time of the signal light field. Otherwise the local oscillator acts as a filter and the quadrature measurement involves only the components of the signal that overlap spectrally with the local oscillator. In the actual experiment, we use pulses from a Ti:sapphire laser with a duration of approximately 120 fs as the local oscillator. The local oscillator is then spectrally filtered to a duration of approximately 1 ps, while the coherence times of the signal are on the order of more than 10 ps, which ensures that the pulses are short enough for all signals investigated here.

Next, in order to obtain a phase averaged quadrature distribution one has to choose a certain number of measured quadratures to perform the averaging process, which in turn corresponds to a certain integration time $t_{av}$. For the experiment performed here, the repetition rate of the laser amounts to 75.39 MHz, which corresponds to a delay of 13.3 ns between consecutive pulses. For the signal light fields investigated within this manuscript, the coherence times are much shorter than that, so one may safely assume that each quadrature measurement randomly samples a different relative phase between the signal and the local oscillator. Under such conditions, it has already been shown that approximately 750 quadrature measurements are sufficient to achieve a sufficient amount of phase averaging^20^. As a result, the analysis yields the mean photon number $n_{av}$ and the value of $g^{(2)}(0)$ of the signal light field within the approximately $10\,\upmu \hbox {s}$ time window needed to perform 750 quadrature measurements.

One may now repeat this experiment for many consecutive time windows. For a typical stable signal, one will get approximately the same mean photon numbers and $g^{(2)}(0)$-values for each time window. However, it is worthwhile to discuss how additional external noise will influence the result. Most external noise sources will arise due to some kind of mechanical noise, which typically occurs at frequencies much lower compared to the inverse of the $10\,\upmu \hbox {s}$ integration time window. Accordingly, external noise will essentially leave the values of $g^{(2)}(0)$ unchanged, but it will modify the measured mean photon numbers. The light field will appear as a light source of constant relative noise properties that shows a slowly modulated mean intensity. We may now quantify this slow photon number noise in terms of

6 $$\begin{aligned} g^{(2)}_{slow}(0)=\frac{\langle n^2_{av}\rangle }{\langle n_{av}\rangle ^2}, \end{aligned}$$

which is the conventional intensity correlation function. This means that Eq. (4) may be used to determine the mean photon number $n_{av}$ within a a time window of $t_{av}\approx 10\,\upmu$s, while Eq. (6) may be applied to quantify the statistics of $n_{av}$ over the full duration of the measurement. Accordingly, the averaging processes in these two equations are very different.

In order to clearly distinguish between the two kinds of noise, in the following we will refer to the intrinsic photon number noise given by Eq. (1) as $g^{(2)}_{fast}(0)$. It is worthwhile to emphasize that typical slow measurements that integrate for seconds or longer will detect both kinds of noise simultaneously. We may now utilize the fact that $t_{av}$ is not a fixed value, but we may choose the integration time freely. As in the experiment each individual quadrature is recorded separately, this choice may be performed after the measurements have already been done. Accordingly, we may analyze the same set of data using different values of $t_{av}$. In doing so, one will find different values of $g^{(2)}_{fast}(0)$ and $g^{(2)}_{slow}(0)$ for each choice of $t_{av}$, which correspond to noise components that occur on faster and slower timescales compared to $t_{av}$, respectively. Accordingly, varying $t_{av}$ systematically provides a thorough analysis of photon noise at different scales.

## Frequency-dependent photon correlations

To demonstrate the possibility to distinguish intrinsic and external noise using ultrafast homodyne detection, we investigate the emission from a non-resonantly excited polariton condensate. The sample is the same one used before^19^ and we used non-resonant cw excitation at the first minimum of the stop band to excite the sample. The excitation beam had a ring-shaped spot shape and a diameter of $12\,\upmu \hbox {m}$. The beam shape was realized using a Holoeye Pluto phase-only spatial light modulator. For each pump power, the local oscillator was resonant with the most intense zero momentum ground state mode, which showed a linear polarization corresponding to one of the crystal axes. A more detailed description of our homodyne detection setup, which is shown in Fig. 1, can be found in a previous publication^20^.

In order to provide context to our experiment and our findings, let us briefly summarize how $g^{(2)}(0)$ of a polariton condensate depends on excitation power in the literature. In most experiments and theories, below the condensation threshold the value of a thermal state $g^{(2)}(0) = 2$ is expected, because here the thermal phonon bath is responsible for polariton relaxation^21,22^. However, this value can only be observed correctly when taking into account the time resolution of the detectors, e.g. of the photo diodes in a HBT setup, see e.g.^23,24^. An exception to $g^{(2)}(0) = 2$ is the case of resonant excitation, where the polaritons can obtain coherence from the pump laser^25^. When increasing the excitation power, $g^{(2)}(0)$ decreases towards a value close to 1 within a narrow range of powers in the threshold region, but then increases again towards a value between 1 and 2, which is roughly constant over a larger range of powers, see e.g.^22,26^. This asymptotic value of $g^{(2)}(0)$ depends on several factors. One factor is spatial confinement, which suppresses the noise induced by different modes to the condensate and therefore enables a smaller value of $g^{(2)}(0)$ nearly reaching 1^24,27^. Similarly, $g^{(2)}(0)$ reaches a smaller value when spatially filtering the emission^28^, because spatial inhomogeneity of the condensate leads to a higher spatial second order correlation^29^. The detuning has an influence on the asymptotic value of $g^{(2)}(0)$ as well^30^. However, in most experiments and theories a value higher than 1 is found due to polariton-polariton scattering^22^ and fluctuations of the reservoir^23^. Finally, when increasing the pump power even further, photon lasing kicks in, which can be observed as a second threshold (depending on detuning) and as a jump in emission energy towards the energy of the bare cavity^31^. Whether this second threshold corresponds to a strong-to-weak-coupling transition or is caused by a non-Hermitian phase transition within the strong coupling regime is still a heavily debated open question^32^.

For the present study, the most important point is that we are always operating at excitation powers above the condensation threshold, but below the second threshold towards photon lasing. We use non-resonant excitation, have no spatial confinement in the planar microcavity and do not filter the emission spatially, except for the spatial overlap with the gaussian shaped LO. Therefore, $g^{(2)}(0)$ is expected to decrease from a value of 2 towards some value between 1 and 2 with increasing pump power, never achieving full coherence. In order to investigate the influence of external noise on the values of $g^{(2)}(0)$ measured in the two regimes mentioned above, we compare two different pump powers. First, we investigate the polariton condensate far above threshold, where the emission is rather stable. Then, we go on to investigate the emission slightly above threshold, where the system response is strongly non-linear and the sensitivity to external noise is strong. In both cases, we compare the sample operating in a standard manner to the sample operating under added mechanical noise which may arise in typical laboratory conditions due to the presence of vibrating pumps or by gently hitting the optical table at one of its edges.

**Figure 2:**
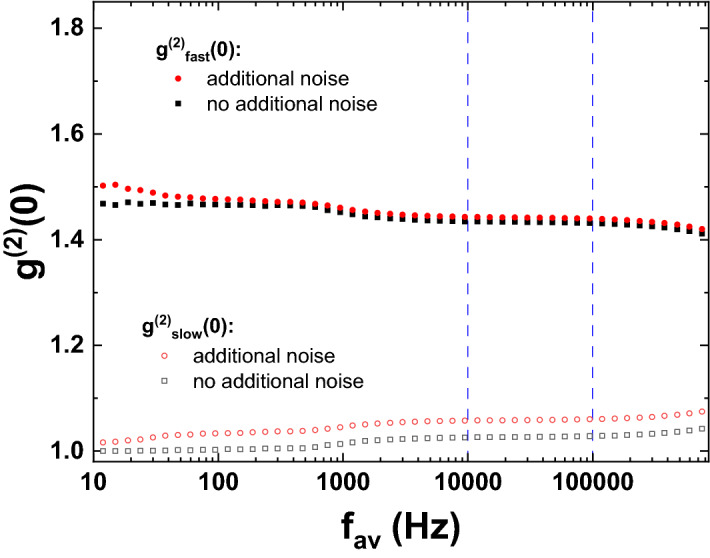
Photon correlation function of a polariton condensate driven far above threshold versus averaging frequency $f_{av}$. Full symbols denote $g^{(2)}_{fast}(0)$, which corresponds to noise contributions at frequencies above $f_{av}$, while open symbols denote $g^{(2)}_{slow}(0)$, which corresponds to noise contributions at frequencies below $f_{av}$. Results for standard operation and operation in the presence of additional mechanical noise are compared. Dashed lines indicate the region, where $g^{(2)}_{fast}(0)$ represents the intrinsic photon number noise of the emission from the sample.

The results for a stable polariton condensate operated far above threshold are shown in Fig. 2 against the inverted integration time

$$\begin{aligned} f_{av}=\frac{1}{t_{av}}. \end{aligned}$$

Black data points correspond to a standard measurement, where only standard environmental noise is present. Red data points represent measurements in the presence of deliberately added mechanical noise. $g^{(2)}_{fast}(0)$, which is expected to correspond to the true intrinsic second-order correlation function of the light field for large $f_{av}$, shows values between 1.43 and 1.5 in both cases. This small range of values shows that the polariton condensate is rather stable against environmental noise for the applied excitation conditions. Still, one may identify three intriguing features. First, the value of $g^{(2)}_{fast}(0)$ reduces further for values of $f_{av}$ beyond 100 kHz. Second, in the presence of additional external noise, $g^{(2)}_{fast}(0)$ shows a significant increase in the low frequency range below 50 Hz. Third, even if solely standard environmental noise is present, one finds an increase in $g^{(2)}_{fast}(0)$ below 1 kHz.

The first effect can be explained easily as an artifact of our detection technique. In order to deduce $g^{(2)}(0)$ correctly from the experimentally determined quadrature values, it is important to ensure that all relative phases between the signal and the local oscillator are sampled equally^15^. As there is no fixed phase between the local oscillator and our signal and the time between two consecutive local oscillator pulses is much longer than the coherence time of the polariton condensate, it is ensured that each measured quadrature is measured at a random phase between the signal and the local oscillator. However, at high frequencies, the number of quadrature measurements used in order to determine the mean photon number and the value of $g^{(2)}(0)$ within a single averaging time window may become quite small. At 100 kHz, this number of measured quadratures amounts to approximately 750. In accordance with our earlier studies^20^, for smaller numbers of measured quadratures and, equivalently, for higher averaging frequencies $f_{av}$, it is not guaranteed that all relative phases are sampled equally. Thus, the results obtained via homodyning are reliable only for $f_{av}$ up to 100 kHz in our setup.

**Figure 3:**
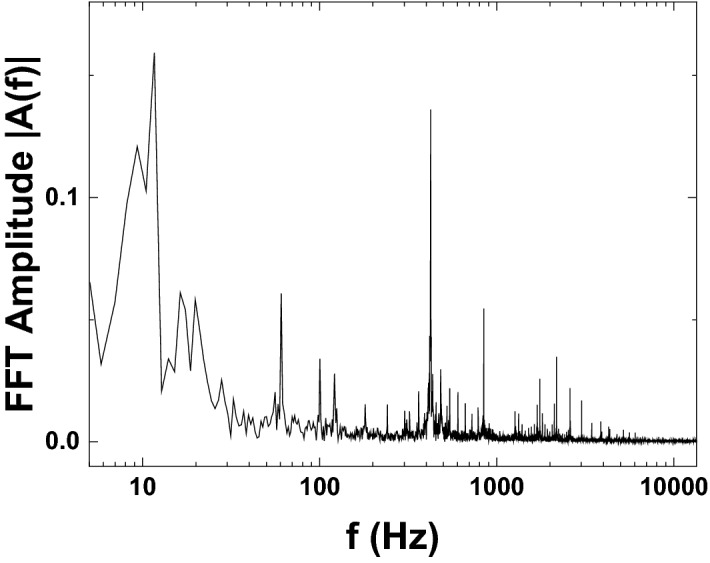
Amplitude of the Fourier transform of the time-resolved intensity emitted from the sample. Continuous low-frequency contributions arise due to mechanical noise, while discrete contributions appearing at multiples of 60 Hz are a consequence of the pulse-width modulation scheme used by the spatial light modulator.

In order to identify the origin of the other two effects, it is worthwhile to compare the values obtained for $g^{(2)}(0)$ versus $f_{av}$ to the Fourier transform of the time-resolved intensity emitted from the sample. A typical frequency-resolved Fourier transform amplitude |*A*(*f*)| in the presence of external noise is shown in Fig. 3.

Here, one can clearly see that the low-frequency noise below 50 Hz corresponds to a continuum of frequencies. It arises mainly due to vibrations and standard mechanical noise. However, in the frequency range above 50 Hz several discrete lines begin to dominate the spectrum, which have an significant influence up to about 1 kHz. The strongest of these lines appears at a frequency of 420 Hz. These lines appear at multiples of 60 Hz and can be traced back to the spatial light modulator used to tailor the shape of the excitation spot. It is a digitally-addressed spatial light modulator, which uses pulse-width modulation of the driving voltage amplitude in order to generate intermediate greyscale values that lie between full and no modulation. To this end, only the dwell time in each of the two voltage states of the digital signal is varied at an addressing frequency of 120 Hz. This modulation scheme results in significant noise contributions at half the addressing frequency and harmonics of that value^33^. This is a common problem of some spatial modulators, which becomes especially important for measurements of photon statistics as this unintended modulation tends to distort the results of experiments. Thus, the increased values of $g^{(2)}_{fast}(0)$ below 1 kHz can be traced back to noise components introduced by vibrations and the spatial light modulator.

The frequency resolved correlation functions clearly show that as $f_{av}$ increases, as soon as it becomes comparable to the frequency of some noise component, the noise component contributions get smoothly redistributed from $g^{(2)}_{fast}(0)$ to $g^{(2)}_{slow}(0)$. These results suggest that in the frequency range between 10 kHz and 100 kHz $g^{(2)}_{fast}(0)$ indeed represents the intrinsic photon number fluctuations of the light field emitted from the sample, while $g^{(2)}_{slow}(0)$ is indicative of external contributions such as mechanical noise. The fact that the deliberate addition of external mechanical noise leaves $g^{(2)}_{fast}(0)$ almost unchanged within this frequency range, while $g^{(2)}_{slow}(0)$ changes considerably, further supports this assumption.

**Figure 4:**
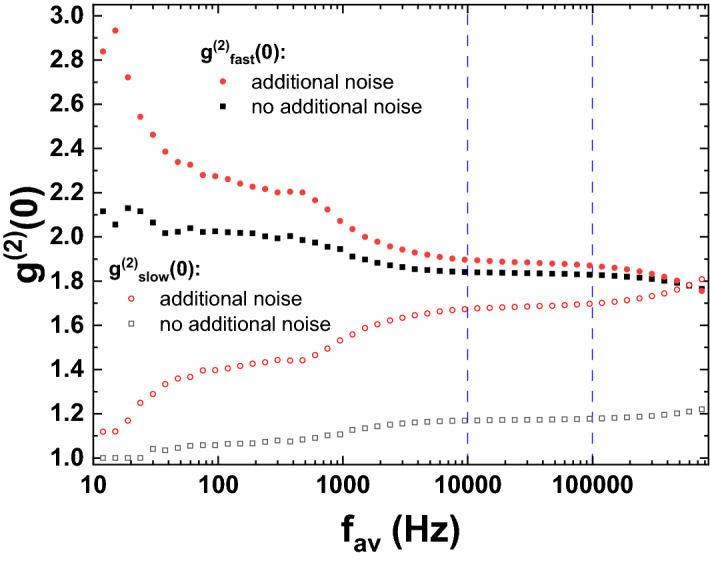
Photon correlation function of a polariton condensate operated at threshold versus averaging frequency $f_{av}$. Full symbols denote $g^{(2)}_{fast}(0)$, which corresponds to noise contributions at frequencies above $f_{av}$, while open symbols denote $g^{(2)}_{slow}(0)$, which corresponds to noise contributions at frequencies below $f_{av}$. Results for standard operation and operation in the presence of additional mechanical noise are compared.

Having ensured that frequency-resolved second-order correlation functions indeed allow us to distinguish intrinsic photon number noise contributions from contributions arising due to external noise sources, we may now investigate the polariton condensate operated within its threshold region, where it is considerably less stable. Figure 4 shows the frequency-resolved photon-correlation for this case. Again, the system is also perturbed by additional mechanical noise for comparison. In the absence of additional noise, $g^{(2)}_{fast}(0)$ changes very little in the relevant frequency range between 10 and 100 kHz and takes on values between 1.83 and 1.84. In the low-frequency range, where photon correlation measurements are usually performed, $g^{(2)}_{fast}(0)$ increases to 2 below 1 kHz and even to 2.1 at very low frequencies below 50 Hz. These results again show that frequency-resolved photon correlations enhance the reliability of the recorded data.

Adding additional external noise enhances the difference between high and low frequencies drastically. Between 10 and 100 kHz $g^{(2)}_{fast}(0)$ shows only a moderate increase compared to the results discussed before and takes on values of about 1.88. The influence of the additional mechanical noise is almost completely absorbed into $g^{(2)}_{slow}(0)$, which increases from 1.17 to 1.68. In the lower frequency ranges, $g^{(2)}_{fast}(0)$ instead increases significantly up to values of about 2.3 and 2.8, respectively. In this case, the results of a conventional photon correlation experiment would not show a close correspondence to the true photon statistics of the emitted light field, anymore.

However, it should be noted that even in the high-frequency range, $g^{(2)}_{fast}(0)$ does not necessarily correspond exactly to the second-order coherence of the emitted light. The slight increase from 1.83 to 1.88 in $g^{(2)}_{fast}(0)$, when the system is subject to noise clearly shows that. In order to explain the origin of this small residual increase in noise, it is worthwhile to discuss the different ways how noise may influence the measured photon number statistics. Most importantly, mechanical noise may change the way how light is collected from the emitter. Mechanical instabilities may slightly perturb the alignment of optics and the overlap of the light field with the detector. In this case, the noise effectively acts as a slow modulation of the mean emitted photon number, which leaves the intrinsic relative photon number fluctuations unchanged. However, as we use optical excitation, mechanical noise may also alter the way how the excitation beam is coupled into the microcavity. In this case, the mechanical noise acts as a slow modulation of the excitation photon number. As in general the photon number statistics of the emitted light will vary with the input photon number, the situation is comparable to a system with a non-linear optical response excited by a noisy source, which is known to exhibit enhanced output photon number noise^34,35^. As this effect corresponds directly to a modulation of the output photon number fluctuations, it cannot be eliminated by investigating different values of $f_{av}$. However, unless the non-linearity is very strong, the change in $g^{(2)}_{fast}(0)$ is rather small. Another kind of noise that can occur apart from intensity noise is polarization noise or spin noise, which has a flat power spectrum up to the order of 100 MHz^36^ and therefore also can not be suppressed by choosing higher $f_{av}$.

**Figure 5:**
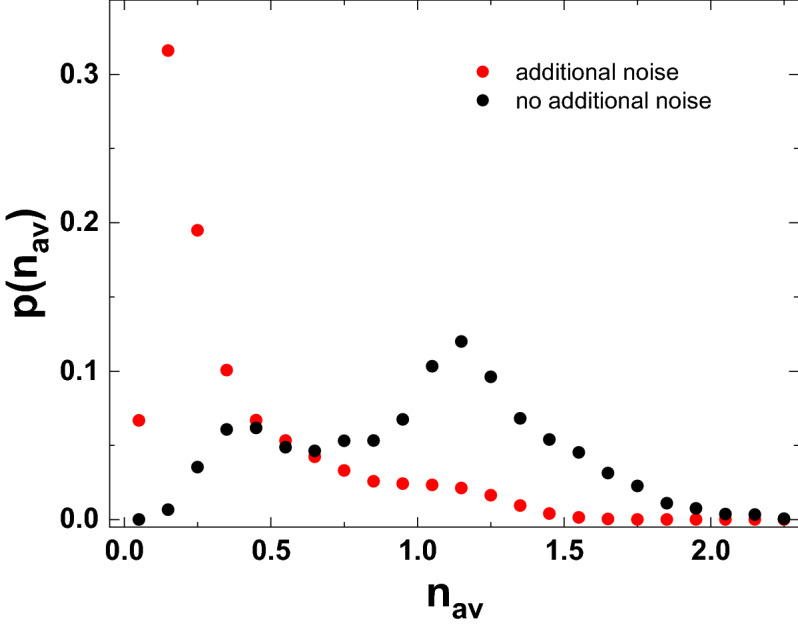
Probability distribution of the mean photon numbers emitted from a polariton condensate ground state during the duration of a local oscillator pulse at an averaging frequency of 75 kHz. Black and red dots denote the distributions in the absence and presence of deliberately added mechanical noise, respectively.

As our experimental setup records each individual measured value instead of histograms, we may apply even more refined statistical tools in order to gain further insights into the system and its response to mechanical noise. In our experiment, we subdivide the full set of measured quadrature values into bunches of a fixed number of consecutively measured quadrature values. Doing so allows us to assign a mean photon number $n_{av}$ to each of these bunches. Choosing a fixed bunch length then allows us to determine the statistical distribution of these mean photon numbers. As we determine the photon number via homodyne detection, it should be noted that the mean photon number corresponds to the mean number of photons present in the signal light field per duration of the local oscillator pulse and the mean is taken over many such pulses. The mean photon number distribution for pumping slightly above the threshold is shown exemplarily in Fig. 5 for a bunch size of 1000 consecutively measured quadrature values per bunch, which corresponds to an averaging frequency of about 75 kHz. The bin width is chosen as 0.1 photons per bin. In the absence of external noise, the photon number distribution shows a double-peak structure with two maxima, where one maximum appears at a photon number of about 1.15 photons. This value corresponds to standard stable emission of the polariton condensate. Another peak of smaller magnitude appears at a photon number of about 0.4, which is mainly caused by the pulse width modulation scheme of the spatial light modulator. During the modulation of the voltage, the excitation spot and intensity change, which puts the polariton condensate below threshold temporarily. Mechanical noise might smear out the photon number distribution further, but seems to be a minor factor compared to the influence of the spatial light modulator under standard conditions.

However, when the mechanical noise present is increased drastically and deliberately, the output photon number distribution changes strongly as is shown by the red dots in Fig. 5. Apart from the added noise the excitation conditions are identical to the experimental data shown before. Now, the photon number distribution shows a dominating peak at low photon numbers below 0.25, which has a long tail towards higher photon numbers and a second small peak at photon numbers close to 1.15, which correspond to standard operation of the polariton condensate. Again, it seems reasonable to attribute this change to modulations of the coupling of the excitation light field to the microcavity caused by the additional mechanical noise as the response of the microcavity to the input light field is strongly non-linear within the threshold region.

## Tracking rare events with postselected photon correlations

**Figure 6:**
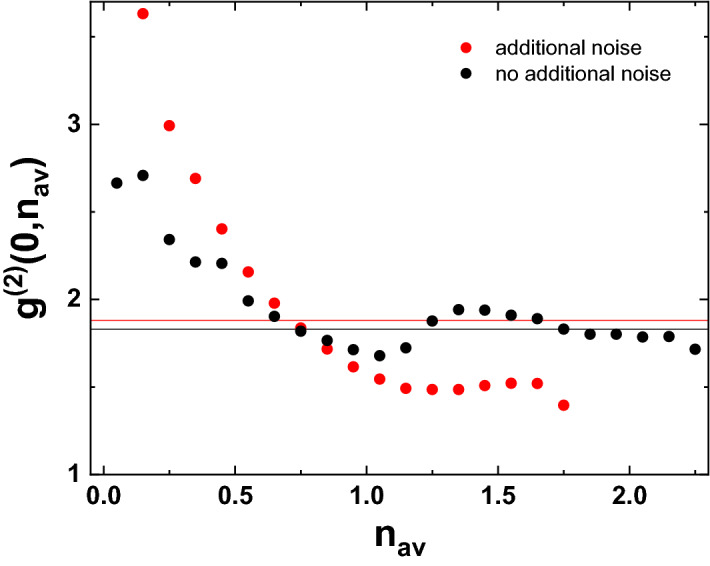
Postselected second-order correlation function $g^{(2)}(0,n_{av})$ for several mean postselection photon numbers $n_{av}$ at an averaging frequency of 75 kHz. Black and red dots denote the correlation functions in the absence and presence of deliberately added mechanical noise, respectively. Solid lines represent the values of $g^{(2)}(0)$ obtained for the same averaging frequency without performing any postselection.

Finally, the full set of information we record makes it possible to obtain further insights into the statistical properties of our system. As we are able to subdivide the whole emission into separate bunches and tag them according to their mean photon number, we may also sort these bunches into freely chosen subsets of interest and determine their emission properties. As an example of what is possible, we investigate the following scenario: As already outlined above, changes in the mean output photon number observed may have different origins. For example, they may be intrinsic to the system under study, they may arise due to modulation of the coupling between the emitted light and the detector or they may arise due to modulation of the excitation light field. In order to estimate the performance and stability of a system, it would be beneficial to distinguish between these scenarios. In order to achieve that, we now use the quadrature measurement bunches sorted into bins according to their mean photon number as used above and determine $g^{(2)}(0)$ for each of these bins individually. Technically, this means that we still calculate $g^{(2)}(0)$ according to Eq. (1), but only pick smaller subsets of quadratures, determined by the mean photon number of the bunches, for the averaging process. Doing so yields a function $g^{(2)}(0,n_{av})$, which yields separate values of the second-order correlation function for each mean photon number. As these values are deduced from quadrature measurements, these are actually values of $g^{(2)}_{fast}(0)$, though we omit the index for brevity. This function can help to distinguish between the different origins of noise. In the case that mechanical noise modulates the coupling between the emitted light and the detector only, the mechanical noise modulates only the effective photon number arriving at the detector at a slow frequency. Accordingly, $g^{(2)}(0,n_{av})$ is not expected to change much with $n_{av}$. On the other hand, if the state of the system changes intrinsically with time or if the mechanical noise modulates the input light field pumping the polariton condensate, $g^{(2)}(0,n_{av})$ is expected to vary with $n_{av}$. Figure 6 shows $g^{(2)}(0,n_{av})$ against $n_{av}$ both in the presence and absence of deliberately added noise. The solid lines show the values of $g^{(2)}_{fast}(0)$ without performing any postselection for comparison. We would like to point out that due to the way it is calculated and also due to the different relative frequencies of occurrence of the different values of $n_{av}$, the value of $g^{(2)}_{fast}(0)$ is not given by simple averaging over the values of $g^{(2)}(0,n_{av})$.

In the absence of additional noise, $g^{(2)}(0,n_{av})$ takes on a minimal value of about 1.68 for $n_{av}$ of about 1.15, which is also the most probable photon number. It seems natural to identify this set of values with the standard steady state of the polariton condensate for the given excitation conditions. When going away from the steady state photon number, $g^{(2)}(0,n_{av})$ shows a significant asymmetric increase. At lower values of $n_{av}$, it rises almost monotonically up to values well above 2. In principle, this increase might be caused by the two possible mechanisms already outlined above: Either the noise modulates the pump beam or the state of the polariton condensate which causes the polariton system to take on a new quasi-steady state that shows larger polariton number fluctuations or the overlap between the emission and the detector is modulated, which only increases the apparent polariton number noise observed at the detector position, but does not really change the steady state of the polariton condensate. While it is not possible to completely disentangle these two effects, comparing the results to the probability distribution for the occurrence of $n_{av}$ already discussed implies that the second peak in that probability distribution observed at mean photon numbers close to 0.4 indeed corresponds to a quasi-steady state of the polariton system below threshold. If the mechanical noise instead modulated the overlap between the emission and the detector, one would rather expect a continuous redistribution of $n_{av}$. Further, $g^{(2)}(0,n_{av})$ takes on values close to the thermal limit of 2 for this subset of $n_{av}$, which further supports the assumption that interpretation that the polariton system operates in a quasi-steady state below threshold for this range of $n_{av}$. For even lower values of $n_{av}$ below 0.3, which are taken on only with low probability, $g^{(2)}(0,n_{av})$ increases to values beyond 2.5, which implies that these subsets of $n_{av}$ correspond to transient states.

Investigating instead values of $n_{av}$ above its most probable value, one finds that for these subsets of $n_{av}$, the postselected correlation function $g^{(2)}(0,n_{av})$ rises up to values slightly below 2 and then levels at values around 1.8, close to the mean value of $g^{(2)}(0)$. As a polariton condensate is a driven-dissipative non-equilibrium system, it seems reasonable to attribute these slightly enhanced values to its non-equilibrium character. Due to the intrinsic polariton number fluctuations, it is not unexpected that polariton occupation numbers above the most probable value occur for limited amounts of time and it is also not unexpected that the system is slightly less stable in these instances as indicated by the enhanced level of fluctuations.

The full benefits gained by a detailed analysis of the postselected second-order correlation function $g^{(2)}(0,n_{av})$ become clear when again comparing the results just discussed to the case of a polariton condensate subject to strong deliberately added noise. As shown by the red dots in Fig. 6, for $n_{av}$ ranging between 0.5 and 1 photons $g^(2)(0,n_{av})$ looks similar to the case without added noise. One may assume that postselection on this range of mean photon numbers mostly corresponds to postselection on instants, where the additional noise does not result in strong perturbations of the system. For lower values of $n_{av}$, however, $g^{(2)}(0,n_{av})$ becomes enhanced significantly compared to the results discussed before and may even show values above 3. This increase can be traced back to the simultaneous presence of the several modulation mechanisms. As shown already in Fig. 5, the probability for such reduced photon numbers to occur is enhanced significantly. This may either be caused by reduced coupling of the emitted light to the detector or by reduced coupling of the excitation light field to the source. Both scenarios will contribute to the detection events within this range of $n_{av}$ and as both result in transient rather than steady states of the polariton system, it seems natural that $g^{(2)}(0,n_{av})$ takes on enhanced values, which reflect that this choice of postselection parameters results in averaging over a broad range of different situations. On the other hand, for values of $n_{av}$ above 1, $g^{(2)}(0,n_{av})$ actually goes down to values on the order of 1.5, which is a value significantly below the noiseless case. While it may seem surprising that adding lots of noise may result in reduced output fluctuations for some postselected conditions, this effect may be explained by the considerations just mentioned. Again, the two main mechanisms that may modify $n_{av}$ are the coupling of the emitted light to the detector and the coupling of the excitation light field to the sample. The first effect may result only in reduced values of $n_{av}$. Assuming reasonable initial adjustment of the optics, adding noise may only reduce the effective detection efficiency. Accordingly, postselection on above-average values of $n_{av}$ implies that effects that reduce the efficiency of coupling the emission to the detector are small or negligible. The influence of modified coupling of the excitation light field to the sample proves to be more complex. In the polariton system, the scattering rate towards the condensate depends on the occupation of the condensate itself, the occupation of the reservoir and the spatial overlap between both. Especially the last point may increase the scattering rate towards the condensate and also influences the number fluctuations of the condensate significantly^37–39^. Accordingly, there is a small chance that noise-induced changes in the excitation light field may actually increase the occupation number of the condensate and put it into a more stable state temporarily. In this case the added noise may act as a temporarily enhanced effective pump rate. This is the case in the regime of postselecting on values of $n_{av}$ larger than 1 and explains the surprising fact that in this range the polariton number fluctuations are smaller compared to the absence of additional mechanical noise.

## Conclusion

In summary, we have demonstrated that homodyne real-time photon correlation measurements open up the possibility to distinguish between intrinsic and external photon number noise by investigating their frequency dependence. As an example, we investigated the emission from a polariton condensate and could show that it is possible to separate intrinsic photon number fluctuations from fluctuations arising due to external influences such as mechanical noise and flicker arising due to optical devices based on liquid crystals. We further showed that our experimental approach makes it possible to perform postselection on the recorded data. As an example, we presented and discussed the counterintuitive result that in rare instances adding strong external noise may actually reduce the photon number fluctuations of the emission from a polariton condensate.

From the spectroscopic point of view, one of the main advantages in the technique we have developed is given by the fact that it yields a clear criterion which allows researchers to determine whether recorded photon correlation data is reliable or not. It further allows researchers to identify different mechanisms that contribute to photon number fluctuations at different frequencies, while it simultaneously preserves a temporal resolution bounded only by the duration of the local oscillator and thus yields access to photon number fluctuations on the sub-ps scale. From the physical point of view, it allows experimentalists to perform postselective spectroscopy, which makes it possible to investigate rare events. We demonstrated this possibility by investigating the fluctuations of a nominally steady state polariton condensate at several postselected instantaneous mean photon numbers.

The detection of some kinds of quantum light sources might be improved by our technique as well. For example, when detecting squeezed light^40,41^, one could yield further insights by analyzing the frequency dependence of noise, as in this case the noise is the figure of merit and each additional noise decreases the degree of squeezing. Another interesting idea is to excite a polariton system with quantum light e.g. from quantum dots and observe the resulting correlations in the polariton emission^42^, where our method also could be applied. In general, every case where noise itself is the desired signal and where a modulation of the mean photon number affects the signal can benefit from our method.

As an outlook, it will be interesting to extend this conditional spectroscopy setup to several detection channels^43^. Doing so will open up the possibility to perform conditional measurements of cross-correlations between several detection channels^44^. For example, it will be feasible to monitor how perturbations of the system relax back towards the steady state by performing postselection on one detection channel and picking the same postselected subset on a second detection channels that measures the emission of the same or a different mode at a later time. Investigating different modes will be especially interesting for multimode systems with nontrivial correlations, e.g. lasers with multiple modes that show gain competition^45^, emitters coupled via a joint carrier reservoir^46^ or systems subject to feedback^47^ and even for molecular aggregates relevant in biochemical processes^48^ . Generally speaking, multichannel homodyne experiments bear great promise for studying steady state fluctuations in optical systems that show dynamics on the timescale of few picoseconds, such as strongly coupled semiconductor systems where frequency-resolved photon correlations are known to carry detailed information about the system^49^.

## Data Availability

The raw data generated during the current study are available from the corresponding author on reasonable request.
